# Rehabilitation, the Great Absentee of Virtual Coaching in Medical Care: Scoping Review

**DOI:** 10.2196/12805

**Published:** 2019-10-01

**Authors:** Peppino Tropea, Hannes Schlieter, Irma Sterpi, Elda Judica, Kai Gand, Massimo Caprino, Inigo Gabilondo, Juan Carlos Gomez-Esteban, Stefan Busnatu, Crina Sinescu, Sofoklis Kyriazakos, Sadia Anwar, Massimo Corbo

**Affiliations:** 1 Department of Neurorehabilitation Sciences Casa Cura Policlinico Milano Italy; 2 Chair of Wirtschaftsinformatik, esp. Systems Development Faculty of Business and Economics Technische Universität Dresden Dresden Germany; 3 Neurology Department, Neurodegenerative Diseases Group Biocruces Bizkaia Health Research Institute Hospital Universitario Cruces Barakaldo, Bizkaia Spain; 4 Ikerbasque: The Basque Foundation for Science Bilbao Spain; 5 Universitatea de Medicina si Farmacie “Carol Davila” Bucuresti Romania; 6 Department of Business Development and Technology Aarhus University Aarhus Denmark

**Keywords:** virtual coaching, rehabilitation, clinical medicine, review, embodied conversational agent, physical activity, health behavior

## Abstract

**Background:**

In the last few years, several studies have focused on describing and understanding how virtual coaches (ie, coaching program or smart device aiming to provide coaching support through a variety of application contexts) could be key drivers for health promotion in home care settings. As there has been enormous technological progress in the field of artificial intelligence and data processing in the past decade, the use of virtual coaches gains an augmented attention in the considerations of medical innovations.

**Objective:**

This scoping review aimed at providing an overview of the applications of a virtual coach in the clinical field. In particular, the review focused on the papers that provide tangible information for coaching activities with an active implication for engaging and guiding patients who have an ongoing plan of care.

**Methods:**

We aimed to investigate the use of the term *virtual coach* in the clinical field performing a methodical review of the relevant literature indexed on PubMed, Scopus, and Embase databases to find *virtual coach* papers focused on specific activities dealing with clinical or medical contexts, excluding those aimed at surgical settings or electronic learning purposes.

**Results:**

After a careful revision of the inclusion and exclusion criteria, 46 records were selected for the full-text review. Most of the identified articles directly or indirectly addressed the topic of physical activity. Some papers were focused on the use of virtual coaching (VC) to manage overweight or nutritional issues. Other papers dealt with technological interfaces to facilitate interactions with patients suffering from different chronic clinical conditions such as heart failure, chronic obstructive pulmonary disease, depression, and chronic pain.

**Conclusions:**

Although physical activity is a healthy practice that is most encouraged by a virtual coach system, in the current scenario, rehabilitation is the great absentee. This paper gives an overview of the tangible applications of this tool in the medical field and may inspire new ideas for future research on VC.

## Introduction

### Background

Virtual coaches are seen as key drivers for health promotion in home care settings. Concurrently, against the background of demographic development, the continuity of care from the professional environment into the home has become critical to ensure patients’ quality of life and to optimize the economics of medical and social treatments. Nowadays, 1 out of 6 people in the European Union (EU) has a disability [1]. Over one-third of people older than 75 years old have disabilities that restrict them to some extent [2]. Acute disease episodes such as stroke, neurodegenerative diseases, heart attack, or diabetes cause most long-term disabilities and entail very costly care processes. In industrialized countries, stroke is the third major cause of death and the most frequent reason for neurological disability [3]. Rehabilitation is a process by which a patient follows a care plan, first in a protected environment (ie, clinic or rehabilitation center) and second at home, the latter case requiring a tutor. The current evidence shows that home-based programs are effective [4], especially in terms of exercise capacity and health-related quality of life, offering comparable benefits to hospital-based programs [5].

As there has been enormous progress in the fields of artificial intelligence (AI) and data processing in the past decade, the use of virtual coaches has gained increasing attention in the considerations of medical innovations. The term virtual coach refers to a coaching program or smart device aimed at providing coaching support through a variety of applications. The virtual coach will be able to personalize and adapt goals according to the progress achieved by the user in their recovery from their impairments or disabilities. It is broadly defined as any form of coaching using electronic media, including or excluding input from a real (human) coach. The virtual coaches guide and train users through a set of tasks, with the aim of supporting positive actions or assisting in learning new skills. For example, virtual coaches could help users to define and preserve a fitness program, suggest problem-solving skills training, or advise patients with specific medical conditions [6-8].

The latest technological developments have shown promise as effective, accessible, and cost-effective solutions. Recent research about the use of embodied conversational agents (ECAs; ie, a computer-generated cartoonlike character, with the ability to produce and respond to verbal and nonverbal communication) has shown that users can efficaciously form a working alliance relationship with a nonhuman agent (ie, a virtual coach) [9].

Virtual coaching (VC) is becoming increasingly important in medical care and in health-related research investments. For example, the recent EU’s Horizon 2020 research framework program funded several projects on VC through a specific call entitled “Personalised coaching for well-being and care of people as they age” [10]. The primary objective of this call was the development of VC solutions that provide personalized advice, guidance, and follow-up support for key age-related issues of daily life that sustain a person’s abilities to remain active and independent (eg, diet, physical activity, risk avoidance, and overall wellness).

### Objectives

Currently, VC for rehabilitation support in the home environment is still in its initial phases of development from the technical, organizational, medical, and ethical points of view. It is of the foremost importance to provide a terminological and conceptual foundation for this new field. Therefore, we sought to investigate the use of the term virtual coach in the clinical field by performing a methodical review of the relevant literature on VC. Pretests of the body of literature for finding a suitable review method revealed a variety of VC approaches, conversational agents and avatar applications, study designs, diseases, and outcome measures, such that the approach of a traditional systematic review appeared inappropriate. We decided to use the scoping review method [11] to map the key concepts behind a research area as well as the main sources and types of evidence that are available [12]. In addition, we wished to underline that, during the investigative process, we focused on coaching activities intended as innovative modalities designed to engage and guide patients who have an ongoing plan of care.

This review can be used as a starting point by clinical researchers and clinicians interested in VC, revealing the potential of VC systems in the clinical domain, and supporting the understanding of the essential components of such interventions.

## Methods

### Search Strategy

We chose to adopt and follow the Arksey and O'Malley scoping review methodology [11]. The literature review was conducted on the PubMed [13], Scopus [14], and Embase [15] databases to find VC papers focused on the medical domain. Studies were collected up to July 2019.

Considering the main key concept for our research, specifically a coaching program or smart device aimed at providing coaching support through a variety of applications, we identified the relevant key words (namely, virtual coach and virtual trainer) for an initial search strategy. These keywords were then refined and used for the identification of related terms. Thus, the final keywords were as follows (syntax was adjusted for each database as necessary): “virtual coach” (OR “virtual coach interventions” OR “virtual coaching”); “virtual trainer”; “virtual training” (OR “virtual training platform” OR “virtual training system”); “virtual therapist”; “virtual nurse”; “virtual agent”; “embodied conversational agent”; “avatar assisted therapy” (OR “avatar mediated training” OR “avatar therapy”); “e coach.”

In addition, wildcard symbols, such as hyphens or inverted commas, were used to consider all possible variations of root words (eg, plural). Limiting categorical area terms were used for the Scopus database: *Medicine*, *Health Professions*, and *Psychology*. Only published journal papers written in English were included, but there was no limitation regarding the publication date.

In addition to the electronic database search, a manual search was performed in JMIR for issues dated up to July 2019. Moreover, we carefully checked references to each article we found to avoid missing relevant papers not identified in the electronic search (namely, handsearching). Two authors (PT and IS) conducted the literature search, and they stored the references in a spreadsheet program, removing duplicates.

### Inclusion and Exclusion Criteria

In a second step, after electronic searching and handsearching, we used the titles and abstracts of the identified articles to check their pertinence to the medical domain. At this step, we selected papers that focused on specific VC activities dealing with clinical or medical contexts, excluding those aimed at surgical settings or electronic learning (e-learning) purposes.

The preselected abstracts were considered for full-text analysis if the related VC had a reasonable sense of agency, with some extent of autonomous behavior able to exhibit some form of reasoning. Furthermore, 3 reviewers (PT, IS, and MCo) independently examined the search results of possible relevant papers. Consensus rounds were used to solve disagreements in the selection.

### Full-Text Assessment

Publications that satisfied the initial inclusion and exclusion criteria were downloaded into bibliography manager software [16] for further screening toward a deeper investigation of the same, previous listed inclusion and exclusion criteria. To meet the objectives of this scoping review, we gathered the following information from each paper: (1) clinical field of application, (2) interface used, (3) study typology, (4) type and number of enrolled participants, (5) intervention duration, (6) study aim or purpose, (7) outcome measurement, and (8) overall conclusions.

Data were summarized in a descriptive form. A quantitative assessment was attempted, but the wide heterogeneity in the study design, instrumental technologies, characteristics of the studied subjects, and analytical methods adopted in the studies prevented any type of meta-analysis of data.

## Results

### Quantitative Results and Overview

**Figure 1:**
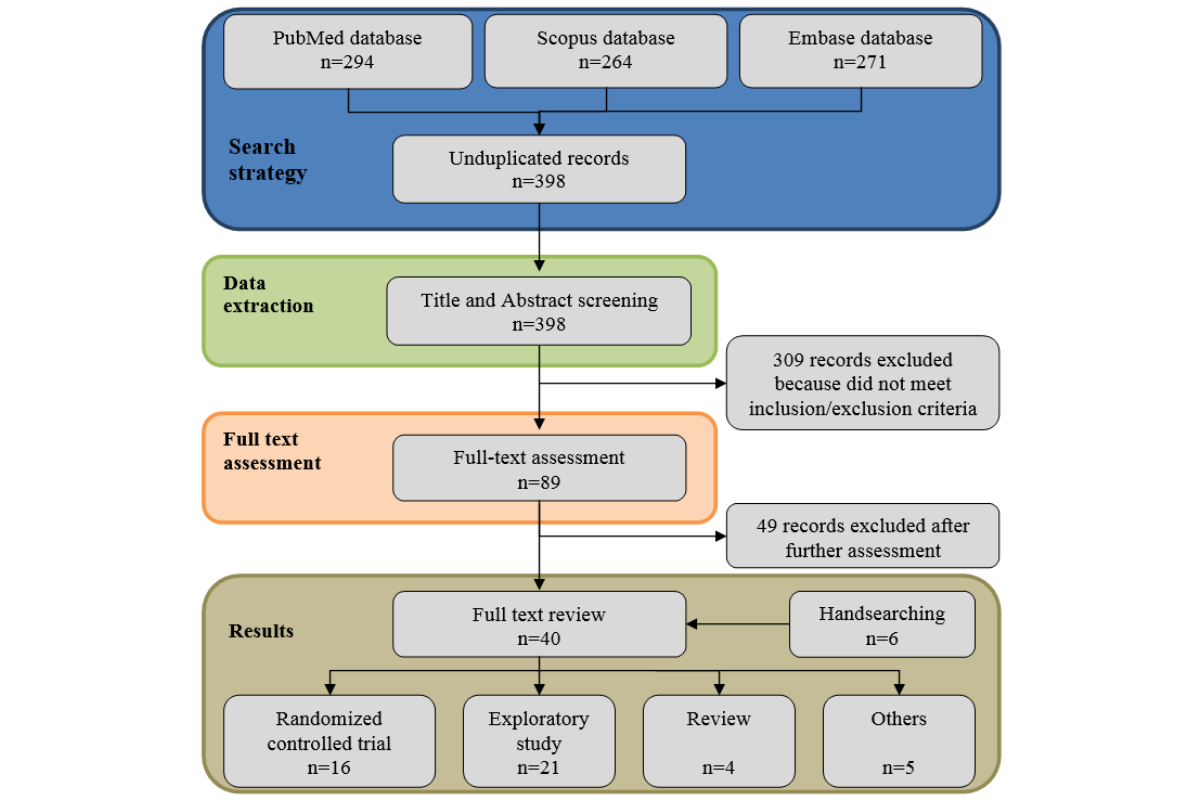
Flowchart describing study's identification and selection.

Figure 1 shows the paper selection process. A total of 294 records were identified in PubMed [13], 264 in Scopus [14], and 271 in Embase [15] databases. Overall, 398 nonduplicate records were identified in the initial electronic search (274 were duplicated, 154 were common to all database, 47 were unique from PubMed, 56 were unique from Scopus, and 19 were unique from Embase).

After reading the titles and the abstracts, 309 records clearly did not meet our eligibility criteria; the 89 remaining records were selected to undergo further analysis. Several articles were related to clinical aspects; however, they were not focused on specific coaching activities. Therefore, we further excluded 49 records that did not meet the inclusion and exclusion criteria for full-text assessment. In detail, 27 papers were excluded because they were related to mere virtual training approaches, without any implication on coaching activities or clinical applications, 8 analyzed e-learning platforms, and 6 were related to emotional aspects of ECA (nondirectly related to clinical interaction). In addition, 3 were excluded because they presented a model of user-interface communication services, 2 were only related to reminder or agenda services, 2 presented the analysis of interactions between users and commercial virtual environments, and 1 was a review on behavioral changes. In addition, using handsearching, we retrieved 6 additional records, 1 of which was a conference paper previously not included by automatic research.

**Figure 2:**
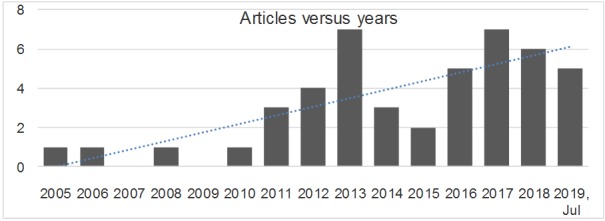
Included articles versus years.

After a careful revision of inclusion and exclusion criteria, a total of 49 records were finally selected to undergo the full-text review. In addition, 16 of the 46 studies selected were clustered as randomized controlled trials (RCTs), 21 as exploratory studies, 5 as review articles, and 5 as theoretical studies. Figure 2, showing the distribution in time of the selected 46 scientific articles in terms of their publication date (ranging from 2005 to July 2019), reflects a noteworthy increase in interest regarding the use of VC in the clinical setting in the last few years.

Tables 1-5 summarize the main features of the identified scientific articles on VC.

**Table 1:** Studies on virtual coaching applied to clinical or medical contexts included in the analysis as randomized controlled trials.

Paper reference	Clinical field of application	Interface	Subjects enrolled	Subjects (n)	Testing period (in weeks, unless otherwise specified)
Allen et al 2008 [17]	Chronic conditions	Not real-time text message (email)	Chronic pain, depression, or impaired mobility	121	4
Gabriele et al 2011 [18]	Obesity in adults	Not real-time text message (email)	Healthy overweight	104	12
Martorella et al 2012 [19]	Chronic conditions	ECA^a^ (virtual nurse)	Adults scheduled for their first cardiac surgery	60	1 day
Watson et al 2012 [20]	Obesity in adults	ECA with verbal and nonverbal communication	Healthy overweight or obese	70	12
Bickmore et al 2013 [21]	Nutrition and physical activity	ECA (computer-based)	Healthy	122	8
Bickmore et al 2013 [22]	Physical activity	ECA with verbal and nonverbal communication	Healthy elderly	263	8 (follow-up 1 year)
Wijsman et al 2013 [23]	Physical activity	Avatar	Healthy elderly	235	12
Friederichs et al 2014 [24]	Physical activity	Avatar (textual interface)	Healthy	958	8
Vroege et al 2014 [25]	Physical activity	Avatar	Healthy elderly	235	12
Broekhuizen et al 2016 [26]	Physical activity	Avatar	Healthy elderly	235	12
Leahey et al 2016 [27]	Obesity in adults	Periodic email contact	Healthy overweight	75	40
Ritchie et al 2016 [28]	Chronic conditions	Interactive voice and a dashboard for the nurse to review data	Chronic heart failure and COPD	511	12
Gardiner et al 2017 [29]	Health promotion	ECA	Healthy	61	4
King et al 2017 [30]	Physical activity	ECA (computer-based)	Healthy elderly	245	52
Cotè et al 2018 [31]	Chronic conditions	ECA (virtual nurse)	Kidney transplant patients on immunosuppressive medication	70	1 day
Hui et al 2018 [32]	Physical activity	ECA with verbal; face-to-face communication	Healthy	200	12

^a^ECA: embodied conversational agent.

**Table 2:** Studies on virtual coaching applied to clinical or medical contexts included in the analysis as randomized controlled trials.

Paper reference	Study aim	Outcome measurement	Conclusion
Allen et al 2008 [17]	Engage and empower patients to collaborate with their primary care physician in managing their health conditions	Access to Web site; access to personal online worksheets; number of emails sent to the electronic coach	Nurses can play an important role, joining efforts to develop new territory to promote patients as partners in managing their health conditions.
Gabriele et al 2011 [18]	The effects of electronic coach support on weight loss, dietary behavior, physical activity, and engagement	Waist and hip circumstances; questionnaire for intervention engagement; Social Support Inventory; dietary behavior	Nutrition education apps are feasible and acceptable solutions to support health promotion interventions.
Martorella et al 2012 [19]	Investigate the preliminary effects of a virtual nursing intervention to improve pain relief in patients undergoing cardiac surgery	Questionnaires; pain intensity score (at the time of admission and after surgery)	Web-tailored approach can increase accessibility to health education and promote pain relief without generating more costs.
Watson et al 2012 [20]	Increase activity levels, via step count, in overweight or obese individuals	Step count	The virtual coach was beneficial in maintaining activity level.
Bickmore et al 2013 [21]	Promote both physical activity and fruit and vegetable consumption through a series of simulated conversations with users on their home computers	IPAQ^a^; step count; National Institutes of Health - National Cancer Institute (NIH-NCI) fruit and vegetable scan; weight	Automated health intervention software designed for efficient re-use is effective at changing health behavior.
Bickmore et al 2013 [22]	Compare the efficacy of a computer-based physical activity program with that of a pedometer control condition in sedentary older adults	Daily step count	An automated exercise promotion system deployed from outpatient clinics increased walking among elderly over the short-term.
Wijsman et al 2013 [23]	Assess whether a Web-based intervention increases physical activity and improves metabolic health in inactive older adults.	Wrist activity monitor; anthropometric measures; blood samples analysis	In inactive older adults, a 3-month Web-based physical activity intervention was effective in increasing objectively measured daily physical activity and improving metabolic health.
Friederichs et al 2014 [24]	Determine whether a Web-based physical activity intervention based on motivational interviewing with an avatar results in more positive appreciation and higher effectiveness of the intervention, when compared to an intervention that is purely text-based	Web-based questionnaires; IPAQ	Avatars that do not strengthen the social relationship with the user do not enhance the intervention impact.
Vroege et al 2014 [25]	Assess how many participants successfully reached the physical activity level as targeted and the effects of the intervention on body composition and metabolic health	Wrist activity monitor; body mass index; blood samples analysis	Of the intervention group, 42.0% reached their daily physical activity end goal, which was associated with a markedly better effect on body composition and metabolic health compared to the effect in the entire intervention group.
Broekhuizen et al 2016 [26]	Assess if an internet-based intervention aimed to increase physical activity was effective in improving quality of life of inactive older adults	Wrist activity monitor; Dutch paper version of the Research and Development 36-item health survey	Internet-based physical activity program was effective in improving quality of life in 60-70-year-olds after 3 months.
Leahey et al 2016 [27]	Efficacy of a novel approach to weight loss maintenance based on modifying the cost–benefit ratio	Weight	Internet delivered cost–benefit approach to weight loss maintenance may be effective for long-term weight control.
Ritchie et al 2016 [28]	Evaluate the impact of a technology-supported care transition support program on hospitalizations, days out of the community and mortality	Number of rehospitalizations, death, days in the hospital, and out of the community	Clinically meaningful reduction in 30-day rehospitalization rates in chronic obstructive pulmonary disease patients when using an interactive voice response–enhanced care transition intervention.
Gardiner et al 2017 [29]	Evaluate the feasibility of using an ECA to teach lifestyle modifications to urban women	Patient Health Questionnaire; Household Food Insecurity Access Scale; Self-Efficacy for Exercise Scale; Perceived Stress Scale	It is feasible to use an ECA to promote health behaviors on stress management and healthy eating among diverse urban women.
King et al 2017 [30]	Compare the effectiveness of 2 linguistically and culturally adapted, community-based physical activity interventions with the potential for broad reach and translation	Changes in walking and other forms of physical activity measured via self-reporting and accelerometers.	The intervention has substantial potential to reduce the health disparities gap by influencing a key health behavior in underserved populations.
Cotè et al 2018 [31]	Evaluate the acceptability, feasibility, and preliminary efficacy of virtual nurse intended to support medication adherence among kidney transplant recipients	Web-Based Nursing Intervention Acceptability Scale; number of completed sessions; medication adherence measures	The results support the feasibility and acceptability of proposed virtual nurse. It could constitute an accessible adjunct in support of existing specialized services.
Hui et al 2018 [32]	Evaluate the effectiveness of an information technology-based lifestyle intervention program on improving physical activity level and health status in a sample of middle-aged Hong Kong adults	Physical Activity measured by accelerometer; IPAQ	The information technology–based lifestyle intervention is fast, inexpensive, flexible, and convenient for adults, especially those with a busy work life.

^a^IPAQ: International Physical Activity Questionnaire.

We identified 5 reviews on VC. To define the fundamentals and elementary components of the term virtual coach, we first analyzed the reference paper by Ding et al [53]. Recently, another review examined electronic health (eHealth) interventions with the combined use of self-tracking and persuasive electronic coaching (eCoaching) [54]. In the psychiatric field, 2 reviews were focused on the use of ECA in psychotherapy [55] and the use of conversational agents for benefits in psychoeducation and self-adherence in the screening, diagnosis, and treatment of mental illnesses [56]. Another recent review identified some technological systems that could rehabilitate elderly patients with insomnia, including virtual coaches [57].

Regarding their clinical fields of application for VC, most of the identified articles classified as RCTs directly or indirectly addressed the topic of physical activity [22-26,30,32]. Some RCTs were mainly focused on the use of VC to manage being overweight [18,20,27] or nutritional issues [21] instead of physical activity. Other RCTs dealt with technological interfaces to facilitate interactions with patients suffering from different chronic clinical conditions such as heart failure, chronic obstructive pulmonary disease (COPD) [28], depression and chronic pain [17] or therapy adherence [19,31]. Similar usage contexts, namely physical activity [33,34,37,40,42,48], nutrition [6], and chronic condition [35,39,41,43,47,51,52], were also found in most exploratory studies. Finally, we also identified other interesting exploratory studies that evaluated the psychological effects [36,44] and the psychoeducational benefits of VC for the users [38,45,46].

**Figure 3:**
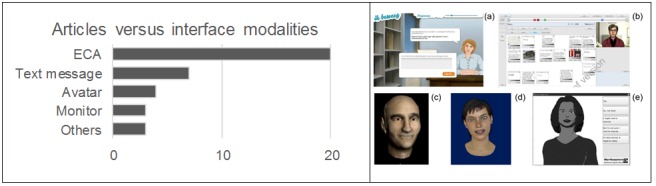
On left: number of studies (randomized controlled trial or exploratory) versus interface modalities. On right: some examples of embodied conversational agents (ECAs) reported in the selected articles: (a) Friederichs et al 2014, (b) Tielman et al 2017, (c) Stevens et al 2016, (d) de Rosis et al 2006, Novielli et al 2012, (e) Bickmore et al 2013a, Ellis et al 2013.

**Figure 4:**
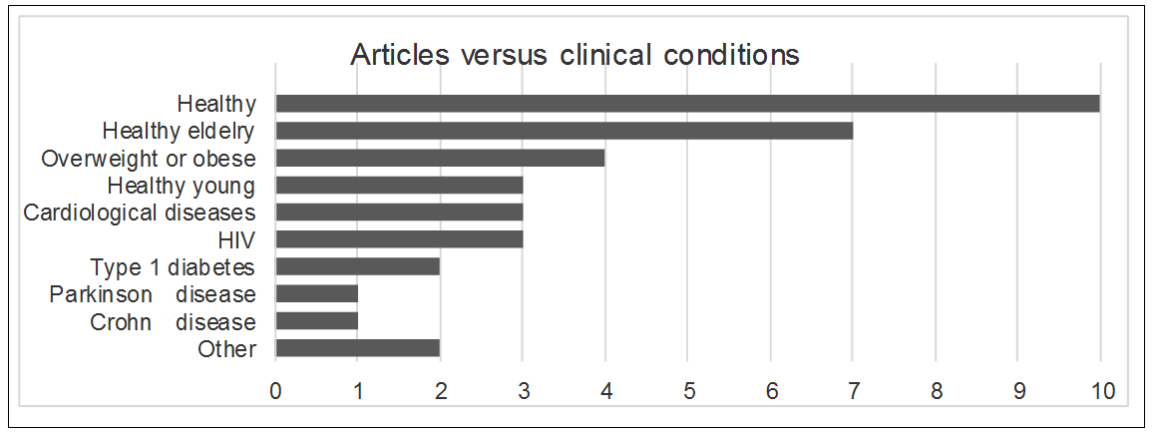
Number of studies (randomized controlled trial or exploratory) versus clinical conditions.

Conversely, we identified several scientific articles that highlighted the growing interest in the use of computer-based ECAs for clinical purposes, both in the context of physical activity [19-22,30-32,35,40,41,43,51] and psychological applications [36,38,42,44,45,49] (Figure 3). Moreover, the ECAs that simulated the key features of a human conversation have also been the subject of a scoping review centered on clinical psychology [55] and 5 theoretical articles focusing on their modeling and architecture [58-62]. Figure 4 shows the distribution of the clinical conditions treated in the 46 studies.

**Table 3:** Studies on virtual coaching applied to clinical or medical contexts included in the analysis as exploratory studies.

Paper reference	Clinical field of application	Interface	Subjects enrolled	Subjects (n)	Testing period (in weeks, unless otherwise specified)
Guillen et al 2005 [33]	Physical activity	Touch screen monitor	Healthy	30	8
Segerståhl et al 2011 [34]	Physical activity	Not real-time text message (Web-based)	Healthy	30	3
Cotè et al 2012 [35]	Chronic conditions	ECA^a^ (virtual nurse)	HIV positive	71	1 day
Novielli et al 2012 [36]	User’s reactions	ECA with verbal; face-to-face communication	Healthy young	30	Variable
Ellis et al 2013 [37]	Physical activity	ECA (computer-based)	Parkinson disease	20	4
Hudlicka 2013 [38]	Meditation Training	ECA	Healthy	32	3
Kreps et al 2013 [39]	Chronic conditions	SMS text reminders	Crohn disease	30	35
Silveira et al 2013 [40]	Physical activity	ECA with verbal and nonverbal communication (tablet based)	Healthy elderly	44	12
van Vuuren et al 2014 [41]	Chronic conditions	ECA with verbal and nonverbal communication (virtual therapist)	Chronic aphasia resulting from a single left hemisphere stroke	8	9
Adams et al 2015 [42]	Physical activity	Not real-time text message (email) and phone call	Coronary artery bypass grafting	1	16
Cotè et al 2015 [43]	Chronic conditions	ECA (virtual nurse)	HIV positive	26	—^b^
Stevens et al 2016 [44]	User and ECA interactions	ECA (computer-Based)	Healthy young	40	1 day
Tielman et al 2017 [45]	Psycho-education	ECA	Healthy	46	3 days
Chi et al 2017[46]	Social isolation in older adults	Pet avatar (tablet based)	Healthy elderly	10	12
Gabrielli et al 2017 [6]	Nutrition	Mobile app	Overweight children and their parents	6 children 6 parents	6
Klaassen et al 2018 [47]	Chronic conditions	Text messages and notifications to the website and to the application	Adolescents with type 1 diabetes	21	6–8
Oyibo et al 2018 [48]	Physical activity	Video	Healthy	659	1 day
Richards et al 2018 [49]	Urinary incontinence	ECA (Web-based)	Children	74	26
Suganuma et al 2018 [50]	Psychotherapeutic intervention	ECA with verbal; face-to-face communication	Healthy	128	2
Dworkin et al 2019 [51]	Chronic conditions	ECA	HIV positive	43	12
Srivastava et al 2019 [52]	Chronic conditions	Virtual coaching	Patients with prediabetes	10	1 day

^a^ECA: embodied conversational agent.

^b^Missing data.

**Table 4:** Studies on virtual coaching applied to clinical or medical contexts included in the analysis as exploratory studies.

Paper reference	Study aim	Outcome measurement	Conclusion
Guillen et al 2005 [33]	Assess technological platform and the fitness condition.	Heart rate; VO_2_	The coach is based on the personalized training programs adapted to user’s characteristics and preferences and on a continuous assessment of the actual fitness status.
Segerståhl et al 2011 [34]	Investigate how users incorporate a system employing 2 modes of delivery into their training and analyze benefits in personal exercise monitoring.	Heart rate	Personal exercise monitoring systems may be improved by more systematically combining mobile and Web-based functionality.
Cotè et al 2012 [35]	Study of the acceptability and feasibility of a Web application which was designed to empower people living with HIV to manage their daily antiretroviral therapies	Acceptability questionnaires; field notes; observations	The results of the study support the feasibility and acceptability of the intervention.
Novielli et al 2012 [36]	Investigate the user’s reactions to received suggestion by an embodied conversational agent (ECA) playing the role of artificial therapist in the healthy eating domain	Classification of speech; user’s reactions	The adaptation of the dialogue is crucial for an effective persuasive interaction.
Ellis et al 2013 [37]	Feasibility, acceptability, and preliminary evidence of effectiveness of a virtual exercise coach to promote daily walking in community dwelling persons with Parkinson disease.	Six-minute walk test; gait speed; number of steps per day; retention rate; satisfaction; interaction history	Sedentary persons with PD successfully used a computer and interacted with a virtual exercise coach. Retention, satisfaction, and adherence to daily walking were high over 1 month.
Hudlicka 2013 [38]	Develop and evaluate a virtual mindfulness coach for training and coaching in mindfulness meditation	Web-administered surveys 5-point; Likert scale	Virtual coach-based training of mindfulness is both feasible, and potentially more effective, than a self-administered program.
Kreps et al 2013 [39]	Describe how electronic health communication programs can be improved by using artificial intelligence to increase immediacy	Weight; patient’s activity; sleep patterns	Artificial Intelligence can enhance the ‘‘immediacy’’ of eHealth by humanizing health promotion efforts, promoting physical and emotional closeness.
Silveira et al 2013 [40]	Investigate which information technology-mediated motivation strategies increase adherence to physical exercise training plans in older people	Adherence and attrition; gait speed; motivation instruments	The social motivation strategies were more effective to stimulate the participants to comply with the training plan and remain on the intervention.
van Vuuren et al 2014 [41]	Analysis of performance of a system for delivering speech and language therapy to people with aphasia, delivered by a virtual therapist	Performance in terms of word accuracy	For persons with aphasia, receiving treatment in an ecologically valid real-world setting delivered by a virtual therapist that provides more cues than not can lead to faster learning.
Adams et al 2015 [42]	Investigate how modify desired movements and activities in a way that minimizes shoulder joint abduction, extension, and flexion	Blood pressure; amount of work done in weight training	The VC, based on a sport-specific, symptom-limited exercise program, would enable the patient to train at a higher intensity than is typically allowed.
Cotè et al 2015 [43]	Explore and describe how patients living with HIV experience receiving customized asynchronous accompaniment via a virtual nurse	Semistructured interviews to get participants to share their experience of the intervention through personal stories and what they thought and felt during their participation	The virtual nurse humanized the experience and helped them acquire new skills for achieving optimal antiretroviral therapy adherence.
Stevens et al 2016 [44]	Investigate the effect of features of human behavior on the quality of interaction with an ECA	Number of errors in ECA speech; multiple-choice questions	Influences from mimicry can be explained by visual and motor simulation, and bidirectional links between similarity and liking.
Tielman et al 2017 [45]	Study the preferable presentation mode for improving adherence.	Behavioral data; questionnaires	Both the attitude towards the virtual agent and how well the psychoeducation was recollected were positively related to adherence in the form of task execution.
Chi et al 2017 [46]	Examine the perceived acceptance and utility of a tablet-based ECA (termed *digital pet*) for older adults	Semistructured, individual interviews for testing of a digital pet companion	A digital pet can provide older adults with companionship and enhance social interaction.
Gabrielli et al 2017 [6]	Describe the design and development of a nutrition education app and the results of a formative evaluation with families	Knowledge of the Mediterranean diet; URICA (University of Rhoda Island Change Assessment)-short-form scale; intention to use technology for nutrition education	The user-centered design showed that nutrition education apps are feasible and acceptable solutions to support health promotion interventions in primary care.
Klaassen et al 2018 [47]	See how patients with type 1 diabetes experience the game with a VC	System Usability Scale; semistructured interview to explore user experiences	User evaluations with patients under pediatric supervision revealed that the use of mobile technology in combination with Web-based elements is feasible.
Oyibo et al 2018 [48]	Evaluate determinants of bodyweight exercise performance in the context of behavior modeling in fitness apps	Social cognitive theory instruments	The study provides a set of guidelines for the design of persuasive technologies for promoting regular exercise behavior.
Richards et al 2018 [49]	Evaluate a novel approach that involves a website and virtual specialist for patients while they are awaiting their specialist appointment	Cross-cultural continence-specific quality of life; Pediatric Incontinence Questionnaire; ehealth literacy survey (eHEALS) ; Newest Vital Signs test; Working Alliance Inventory	A novel approach that involves a website and virtual specialist for patients while they are awaiting their specialist appointment showed an overall improvement in 74% of patients with urinary incontinence.
Suganuma et al 2018 [50]	Use an internet-based cognitive behavioral therapy preventative mental health measure	World Health Organization-Five Well-Being Index; Kessler 10; Behavioral Activation for Depression Scale	The internet-based cognitive behavioral therapy with the embodied conversational agent can be used in mental health care.
Dworkin et al 2019 [51]	Evaluate the feasibility, acceptability, and preliminary efficacy of an ECA intervention to improve adherence to antiretroviral therapy	Adherence; acceptability; feasibility, pre- versus posthealth literacy; pre- versus post-self-efficacy	The pilot study of demonstrated acceptability and preliminary efficacy in improving adherence in this important population.
Srivastava et al 2019 [52]	Evaluate performance relative to behavior stages associated with long-term behavior modification	Continuity in adherence to the program	The strength of the physician–patient relationship appears to allow people with prediabetes to skip or advance rapidly through behavioral stages in the process of lifestyle modification.

**Table 5:** Reviews and theoretical studies on virtual coaching applied to clinical or medical contexts.

Paper reference	Clinical field of application	Interface	Study typology
Ding et al 2010 [53]	—^a^	Overview of virtual coach interventions	Review
Lentferink et al 2017 [54]	Lifestyle	e-Coach^b^	Review
Provoost et al 2017 [55]	Psychotherapeutic interventions in clinical psychology	ECA^c^ (virtual human characters on computer screens to robots) and communication	Review
Vaidyam et al 2019 [56]	Psychiatry	Conversational agent, chatbot	Review
Salvemini et al 2019 [57]	Insomnia and sleep disorders	Virtual coach	Review
de Rosis et al 2006 [58]	Health promotion	ECA with verbal; face-to-face communication	Theoretical study
Cotè et al 2011 [59]	HIV positive	ECA (virtual nurse)	Theoretical study
Perez et al 2016 [60]	User and ECA interactions	ECA	Theoretical study
Cotè et al 2017 [61]	Chronic conditions	ECA (virtual nurse)	Theoretical study
Fadhil et al 2019 [62]	Health promotion	ECA	Theoretical study

^a^Not applicable.

^b^e-coach: electronic coach.

^c^ECA: embodied conversational agent.

### Definition of Virtual Coach

#### Overview

The term virtual coach was firstly used in the athletic context during the 1950s, in reference to sporting coaches that were leading the teams behind the scenes or over distance. The earliest medically oriented usage of Virtual Coach was in 1997 in an operating room for guiding a nonstandard surgical procedure. Nowadays, the term virtual coach is a specific technological concept referring to *ad hoc* platforms comprised of one or more devices with a dedicated software that are used for training and coaching interventions. VC platforms use several tasks to support the learning of new activities and encourage positive or correct behavior. According to Ding et al, 4 components (ie, self-monitoring, context awareness, coaching strategy, and interface modality) define the space of action of such interventions and address when, how, and what message, feedback, and stimuli to deliver.

#### Self-Monitoring

This could be defined as the observation and recording of a specific activity or a specific condition, related to personal and physical information (eg, activity levels, calorie intake, and weight). Self-monitoring is performed (also in real time) with a wide variety of sensors that allow the tracking of the user performance, triggering messages to the user only when they are necessary and relevant.

#### Context Awareness

This consists of a collection of specific contextual parameters (eg, location, time, identity, and activity) and of the responsiveness to an interruption of a task based on that contextual information. However, many more factors, such as the emotional state of the user and the reason and modality of the interruption, are crucially linked to the responsiveness [63]. Sensor technologies combined with VC algorithms empower a system to improve the identification of contextual elements (eg, location and user activity), optimizing the identification of activity discontinuation by the user and delivering prompt messages in appropriate moments.

#### Coaching Strategy

A coaching strategy selects which kind of content should be contained in each message delivered to the user, distinguishing general versus specific messages and the incorporation of proper emotional effects within them. The strategy also aims to perform a dynamic adaptation of the activities to the needs, performance, and improvements of the user, which are collected through the two self-monitoring and context awareness phases. Due to the adaptation, the annoying effect could be avoided and the user may be more confident in their use of the system. Moreover, positive messages may help to reinforce the performance, comfort, and interest in using the system. Conversely, the use of negative feedback and messages should be limited as they are normally inappropriate and the aim is to decrease errors.

#### Interface Modality

This includes the different kinds of interfaces (ie, visual, auditory, and tactile) that can be used to deliver coaching messages to users. As matter of fact, ECAs have become the most popular interface because of increasing innovations in the field of computer graphics. ECA interfaces could be very different, ranging from simple animated characters to humanlike or animal-like entities, talking animals, or many other varying forms. One of the common trends is to design animated humanlike agents and then characterize them with a great heterogeneity of features, from its gender, aesthetics, and voice to its personality and behavioral patterns.

### Interaction Between Virtual Coach and the User

As previously mentioned, the ability to provide coaching messages in a way that is personalized both to the user and the situation will likely develop a trustworthy relationship between the VC platform and the user, improving the acceptance and effectiveness of the technology. In this context, the interaction between the virtual coach and the user acquires a priority role. From the analyses of the literature, a description of the interaction between VC and users was found as the relevant topic. Several articles examined this theme in depth [18,20,21,36,49,53,55]. The most comprehensive description of the interaction between VC and users was proposed by Watson et al [20]. In their study, they suggested several steps to organize VC-user communication as a structured process: (1) opening with salutation and social contact; (2) proceeding to review of data acquired, feedback, and goal setting; (3) advice about specific topics (eg, activities and diet); (4) guarantee to time of next contact; and (5) ending with encouragement and farewell.

Moreover, the structure and format of the dialogue, together with the content of individual expressions, needs to be personalized according to the users’ progress in the system, their current status, and the speech context [20]. An acceptable interface for communication should be able to distinguish the communicative and social attitudes of users, and adjust the dialogue and the interaction style accordingly [36]. Due to this personalization, individuals who did not reach their proposed objectives or who had a different attitude towards the communication would have had a different interaction than those who met their goals. The ability to provide personalized feedback and support, in an interactive manner, was the most successful coaching feature [38].

According to Kreps et al [39], the success of health communication interaction depends upon multiple factors, including the accuracy, timeliness, fidelity, persuasiveness, and sensitivity of messages exchanged. In addition, the immediacy is a critical factor in determining whether communication processes work and could help users to work together to achieve important health goals [39].

A group of 20 out of 46 revised papers (see Tables 1 and 3 and Figure 3) used the human ECAs as interface modality of VC. Provoost et al [55] published a scoping review about the use of the ECA in the field of clinical psychology. The authors defined the concept of an ECA via the classification of the roles, characteristics, and skills of these interfaces. Thus, the ECA consists of 3 components:

1. the interface that allows users to communicate with the ECA (eg, from Web-based questionnaires to real-time audio and video input);
2. the algorithm that consists of the mental capacities of the ECA, allowing the ECA to react empathically with users;
3. the so-called embodiment (or visual representation) of the ECA, meaning how it appears (eg, humanoid, toylike, photograph, or fantasy). The look of the ECA is also defined by the way in which it communicates with users (eg, verbally vs nonverbally, text messages vs speech, and with or without gestures and facial expressions).

The authors also categorized the way in which information was conveyed: ECA and users could communicate with each other through speech, facial expressions and eye gaze, hand and body gestures, or simply text [55]. The negative experience performed by Friederich et al [24] corroborated the relevance of considering the multiple verbal and nonverbal communication modalities when designing ECAs. In the cited paper, the avatar, with limited relational skills and unable to respond in gestures to the user’s state and input, was not able to enhance the intervention and therefore did not increase the intervention’s impact.

Simulating facial expressions and eye gaze (face-to-face conversation) is particularly important in health care when dealing with users with low literacy because in those cases, face-to-face encounters with health providers remain the most effective interaction modalities [21]. Regarding the aesthetical characteristics of the ECA, it is important to state that the level of trustworthiness is increased by the personification of the agent. The most recent systems incorporate bots, games, and simulated environments to talking/thinking/teaching ECAs, allowing them to understand the effect of different features of the system on human acceptability, understanding, and learning [44]. The effects of mimicry on likability in human-ECA interaction were tested to investigate whether visual cues displayed appropriate signals than those in the no-mimicking condition. Similarly, the effect of the ECA’s facial gestures (ie, smiling, winking, and rolling eyes) was investigated in a parallel experiment to understand whether comprehension of a story was increased when recited by an ECA with facial expressiveness [44]. Expressive facial gestures may aid learning through increased redundancy and increased cognitive load. In contrast with this, it is reported that some users experienced a phenomenon referred to as the uncanny valley [64], where the general trustworthiness of the agent negatively correlates with the degree of visual realism of the ECA form. In other words, ECAs that are more cartoonish are perceived as more acceptable and effectively realistic than more visually realistic ones [38].

Regarding the social aspects of ECAs, it is worth mentioning that one of the selected articles described 4 social roles for ECAs [55]: (1) the social interaction partner aims to engage in an interaction with the user to improve specific social skills, (2) the tutor aims to teach something, (3) the coach aims to motivate and engage the user, and (4) the health care provider aims to simulate the behavior of a health care provider.

In addition, a pilot study evaluated how older adults could interact with a pet avatar [46]. This study examined the perceived acceptance and utility of a tablet-based conversational agent in the form of a pet avatar that was used by older adults over 3 months ,during daily interactions. The results disclosed that this interface (ie, a digital pet) could provide older adults with companionship and enhance social interaction.

In conclusion, studies indicate that ECAs have the following benefits:

1. Social interaction: relations between a human being and a machine are social; an avatar supports contact in a natural way, like with other human beings.
2. User attention: animated characters can attract the user’s attention.
3. Real simulation: embodied relational agents provide the illusion of liveliness and interacting with a real person.
4. Body language information: facial expressions of the avatar contain a lot of information.
5. Trustworthiness and believability: the level of trustworthiness is amplified by the humanization of the ECA; a realistic face seems to be rated as more intelligent, engaging, and likeable.

### Medical Care Sectors

#### Summary

The analysis of the literature showed that the application of the VC has involved different areas of the medical domain, which allowed us to address the problem from different angles. In particular, the studies evaluated specific topics, such as physical activity and nutritional regimen, or wider contexts linked to the reduction of the risk of illness. The following are the main topics covered.

#### Lifestyle: Physical Activity

The physical activity of adults and elderly people is increasingly recognized as an essential lifestyle behavior in maintaining health and preventing diseases. Therefore, it is not surprising that many articles have tried to introduce an automated exercise promotion system to support motor activity in healthy or overweight elderly subjects.

At the beginning of this decade, Segerståhl and Oinas-Kukkonen [34] supported the idea that physical exercise monitoring systems could be improved by combining mobile and Web functionality. So, they conducted a qualitative field study with 30 participants using a heart rate monitor and a Web service in their training for 3 weeks. Despite the limitations of the study because of the lack of a control group, the authors showed that a system employing multiple modes of delivery may influence personal exercise monitoring as perceived by users, and highlighted the importance of future developments of intelligent coaching that can provide useful and personalized information. Indeed, keeping an optimal physical status is a critical issue in an ageing society. Hui et al [32] tested the effectiveness of a Web-based virtual training system with ECAs to improve physical activity in a sample of middle-aged adults. The system was flexible and convenient for adults, especially for those with a busy work life. In a preliminary validation study, Fadhil et al [62] discussed the outcome of a 1-month use of a conversational agent–assisted health coaching system designed to support health intervention delivery to individuals or groups, showing initial promising results in the engagement and adherence of users and the role of a conversational agent in delivering health-promoting activities.

Among the elderly population, Bickmore et al [21,22] evaluated the efficacy of an automated intervention with the ECA to improve physical activity and fruit and vegetable consumption in sedentary older adults, showing an effective result at changing health behavior with this computer-based program. Regarding physical activity, results of the RCT demonstrated that participants who received the ECA intervention walked significantly more steps, recorded by a pedometer, than control participants at 2 months [22]. In particular, the authors highlighted 2 fundamental factors on the success of physical activity promotion: the importance of face-to-face conversation, using voice, between the ECA and the participants, and their level of health literacy. A subsequent work by Friederich et al [24] confirmed that the relational skills of the avatar are very important in strengthening the relationship with the user and may enhance intervention impact. In fact, they developed a Web-based physical activity intervention based on motivational interviewing that was done by an avatar positioned behind a desk in a small office (Figure 3, on right). The avatar communicated through text balloons, without the use of voice or nonverbal expressions such as empathic gestures and eye and head movements. Consequently, the avatar used in the study was not able to increase intervention effectiveness and the authors concluded that the action of a virtual coach with more complex relational skills had to be recommended for future research. In this context, King et al [30] conceived a study design to evaluate the comparative effects of physical activity recommendations by humans or computers in unserved populations. The authors, confident of the results obtained by using a virtual advisor (ie, ECA) in a preliminary study [65], hoped to obtain, through a comparative effectiveness trial, the confirmation that this program could offer a substantial potential to reduce the health disparities gap by influencing a key health behavior in underserved populations.

In addition, a Web-based physical activity intervention was proposed to increase health and reduce risk factors in the elderly population. In several studies from the same group [23,25,26], an internet program aimed to increase physical activity and improve quality of life of inactive older adults using monitoring and feedback by accelerometers and digital coaching. The authors found that an internet-based physical activity program was effective in improving quality of life after 3 months, particularly in participants that reached their individually targeted increase in daily physical activity. More importantly, they also observed an improvement of objectively measured daily physical activity and metabolic health after a 3-month, Web-based, physical activity intervention. Such Web-based interventions provide new opportunities for large-scale prevention of chronic and metabolic diseases in the aging population.

Similarly, in a preclinical exploratory trial [40], an information technology–based training app that runs on tablets and monitors older people was used to follow personalized training plans autonomously at home, and to improve balance and strength in 44 older adults. The app seemed to assist and motivate the subjects to autonomously perform strength-balance exercises and increase their gait speed. The social motivation strategies were more effective at stimulating the participants to comply with the training plan and lowered the dropout rate.

#### Lifestyle: Nutrition and Obesity

Watson et al [20] demonstrated a sustained level of activity in overweight adults provided with a VC in addition to a pedometer and Web-based feedback, compared with a decline seen in those without VC. Even the use of an electronic coach (e-coach) to support and help users has resulted in them keeping their weight under control [18,27], or the development of an intelligent virtual agent (ie, ECA) to persuade people to improve their eating habits [36] has provided promising results. Limited experiences are documented in the context of nutrition education apps, which however seem to be potentially useful in providing effective prevention and health promotion programs for overweight children [18].

To provide guidelines for the design of persuasive technologies for promoting regular exercise behavior, Oyibo et al [48] conducted an empirical study on 659 subjects to uncover the determinants of the performance of bodyweight exercise behavior. After modeling 2 popular bodyweight exercise behaviors using a virtual coach, they showed that perceived self-efficacy and perceived social support are the strongest determinants of this exercise behavior.

In a cohort of 10 patients with prediabetes, Srivastava et al [52] recently offered a module consisting of a Web app supporting diabetes prevention education and a mobile app with an electronic diary and virtual coach. Thanks to an efficient review of user performance and the ability to send support notifications from the user’s coach or physician, the module reached a high success rate (60%), allowing patients at high risk for diabetes to be engaged in the process of lifestyle modification.

#### Chronic Diseases

VC could play a fundamental role for people with chronic diseases, providing them with support for the self-management of their problems at home. This explains why some authors have begun to evaluate the use of e-coaches in communities of no longer healthy subjects. In 2008, Allen et al [17] developed an internet-based health coaching intervention to enhance patient-provider communication regarding 3 common conditions: chronic pain, depression, and impaired mobility. This efficient and low-cost approach offered an innovative opportunity to improve patient and clinician partnerships in managing chronic conditions. An internet-based health coaching intervention that can offer a significant benefit to many patients, but differs substantially from usual nursing care, was also implemented to empower patients toward collaborative self-management. Providing and evaluating the experience gathered within a community of patients with Crohn disease, Kreps et al [39] showed that VCs and comfortable human-computer interfaces (based on user-centered AI approaches) could stimulate active information processing and adoption of new thoughts, such as motivation and behavior changes. A proof of concept of a pervasive gamified platform framework that is a combination of integrated sensors (wearables), a VC, and serious games in health care has been recently reported by Klaassen et al [47]. Interestingly, they combined different technology components to promote positive health behaviors among young people with type 1 diabetes.

Ritchie et al [28] conducted a study on the use of the system and readmission outcomes of a pragmatic randomized trial of e-coach (as the VC was called in this paper). In the study, the system was used in a diverse sample of complex medical patients with congestive heart failure and COPD from a wide geographic region in the Southern United States. Although 30-day rehospitalization rates did not statistically differ between the e-coach and usual postdischarge care groups, in the COPD subgroup the e-coach was associated with significantly fewer days in the hospital. This indicated that interventions may need to be disease-specific to decrease rehospitalization rates and to ensure adequate postdischarge care. This study addressed the problem of how to manage care transition from the hospital to the home for people with two different critical illnesses. E-coach, supported by an interactive voice response (IVR) system, did not reduce the rehospitalization of these subjects. This evidence led the authors to conclude that, to date, a combination of an IVR and personal contact with the treating physician is the solution to that has the most beneficial effect on reducing preventable readmissions and providing optimal transitional support for these complex patients. In the cardiological field, Adams et al [42] described a single case of a patient who was a powerlifter and returned to his sport after coronary artery bypass grafting with long-distance coaching by the cardiac rehabilitation staff. Through high-intensity training that was complemented by phone and email support, he lifted heavier loads than he had before the bypass grafting.

Regarding the neurological diseases, Ellis et al [37] conducted a phase 1, single-group, nonrandomized clinical trial in Parkinson disease (PD). The intervention was designed to increase the physical activity of 20 subjects with mild-to-moderate PD by promoting additional daily walking using a pedometer and brief daily interactions with the computer-animated virtual exercise coach. Patients successfully used the virtual coach at home over a 1-month period, demonstrating the initial feasibility of this approach.

With a virtual therapist designed for speech therapy that works across devices, and built-in Web monitoring, scheduling, and communication technologies, van Vuuren et al [41] recruited 8 participants with chronic aphasia to receive intensive computer-based script training differing in the amount of high or low cuing provided during treatment. With the limitations of a small sample size and the lack of separation of auditory and visual cuing, the overall effect size for the computerized treatment was large and like what would be expected when treatment was delivered by a speech-language pathologist.

With the role of virtual nursing avatars (VNAs) considered to be promising and evolving [66] in the context of chronic disease, Cotè et al [35,43,59,61] promoted the application of VNAs through a Web app to support the management of antiretroviral therapy and the adoption of healthy behaviors in HIV patients.

In a 3-month pre- to postdesign pilot study, the promotion of HIV medication adherence was also explored in 43 patients using a theory-based, mobile-delivered, ECA to provide information and behavioral skills. They showed good acceptability and preliminary efficacy in improving drug adherence [51].

The promotion of therapy adherence in other chronic diseases was also described through a Web-based nursing intervention in some pilot RCTs [19,31], whose results support the feasibility and acceptability of the virtual nursing sessions in a small sample of patients.

#### Psychology

The use of VC has also involved the field of, both in offering new educational approaches or psychotherapeutic support, and in trying to understand the relationship between ECA and user. In 2013, Hudlicka [38] reported that the virtual coach–based training of mindfulness was potentially more effective than a self-administered program, including written and audio material, in a group of students initiated to this practice. Although it had a low number of participants, the study highlighted the possible effectiveness of virtual training and coaching in ending or acquiring a behavior that may be deleterious (eg, smoking) or healthy (eg, exercise) in people exposed to medical risk. A virtual agent was also used with good results in favoring adherence to a therapeutic task within a psychoeducational process [45]. However, the study showed that if psychoeducation was presented in the text, it resulted in better adherence than if the agent offered it verbally. Indeed, we know how important the mimicry and the expressiveness of the ECA are for its effective interaction with the user. These 2 aspects have been addressed in 2 interesting experiments reported in a recent article by Stevens et al [44] (see “Interaction between virtual coach and the user”). Recently, ECA applications in clinical psychology have been reviewed but the authors concluded that these applications are still limited [55].

#### Risk Factors Reduction

ECAs and VC services with an integrated cognitive training system, interactive clinical scales and tests to identify chewing and swallowing difficulties, with integrated pedometers and other wearable sensors (fall, freezing, or motor fluctuation detection systems), as well as a home-based interactive information environment can be used to identify and prevent disease-related complications at home or outdoors. This type of coaching system has been demonstrated in health problems such as obesity and a sedentary lifestyle [20,22,27]. This user interface would also provide daily motor, cognitive, and behavioral exercises and tips, monitor lifestyle and take care of treatment adherence monitoring, with AI to solve basic inquiries, and would provide contact with urgent or nonurgent health care responses if needed. The hardware of this system would be a combination of home-based wall microphones, speakers, and screens and a mobile phone–based system. A comparable system for heart failure was implemented for the My Heart IST-2002-507816 project [33]. This system was designed to be remotely supervised by humans specialized in health care, such as nurses, providing an environment for transitional care of patients. This would have had an additional positive impact on the prevention of hospital admission–related complications and in reducing sanitary burden and costs by minimizing admission days in the hospital and out of community [21].

#### Treatment Adherence

Any implemented VC service such as ECA should also take specific care of treatment adherence monitoring. In this sense, the creation of a virtual social environment may include that patients could exchange information with other patients or could directly contact their reference health care professionals for treatment-related inquiries [27]. However, the adherence to the treatment or intervention very much depends on the humanity of the ECA, such as showing empathy, emotions, or using human-like speech. Those devices that are monitored and controlled remotely by humans have been shown to be particularly effective [30,46]. Kreps and Neuhauser demonstrated that it is possible to provide computer-mediated support to patients to track their medication adherence [39], and this evidence may be very useful in preventing motor fluctuations and dyskinesias in PD patients, where the chronology of treatment is important [37].

## Discussion

### Findings

This scoping review shows that in the last 10 years, there has been a growing interest in implementing VC programs aimed at stimulating and guiding users toward positive behaviors in the various sectors of the health care world. In the review, we evaluated all available evidence on the use of VC, defined a coaching program, and suggested problem-solving strategies helping users to face specific medical conditions. In most cases, a successful VC approach must consist of participant-involving methods, with a focus on the individual patient’s lifestyle conditions. In addition, the review allowed us to map the key concepts currently tied to the users’ or patients’ VC, such as function and symptoms, medication, dietary recommendations, recognition and coping with alarm signals of worsening of the disease, psychological and emotional reactions in connection with the disease, and exercise strategies. The patients’ relatives or other acquaintances can be involved depending on the patients’ needs and requests, and education must be conducted in a combined individual and group-based setting.

Recently, another review examined eHealth interventions with the combined use of self-tracking and persuasive e-Coaching [54]. It is interesting to note that the review provides information on which key components can be effective on health outcomes, usability, and adherence. Although the analysis is relevant for future strategies that combine self-tracking and persuasive e-Coaching, it addresses the topic of the virtual coach differently from our review, which aimed to analyze the context and the medical implications of the interventions carried out rather than the aspects that can influence the effective use of technological solutions. We extended the investigation to the study protocols, the types of interface adopted, and the medical areas of interest, adding a dedicated section for the latter, and we decided to gather this information as it would allow us to assess the state of the VC application related to its degree of use and diffusion in the medical health field.

### General Implications in the Medical Sector

Regarding the medical care context, to date, the use of virtual coach technology–assisted care seems to be more represented but it has mainly been implemented for healthy subjects with the aim of improving their health behaviors and reducing the risk factors for cardio- and cerebrovascular diseases, such as obesity or physical inactivity. On the contrary, only 7 studies considered groups of patients with chronic diseases (ie, depression, heart failure, COPD, PD, Crohn disease, diabetes, and urinary incontinence) with the intent of demonstrating the usefulness of different internet-based health coaching interventions. These were aimed, for example, at improving communication with nursing staff, reducing the rate of rehospitalization of patients, or promoting their daily physical activity.

It is noteworthy that this review found 16 RCT studies among the 46 articles selected for concrete coaching interventions in the medical health field in the last 10 years. However, although this may represent an interesting fact for a topic that has appeared over the last 15 years, it deserves some specific considerations. First, as the majority of RCT studies involved healthy subjects, although often constitutionally overweight or in old age, their primary aim was often to promote healthy behavior. Moreover, with particular reference to this type of intervention on a healthy population, RCT studies have so far preferentially evaluated the use of VC for the purposes of primary prevention [18,21,22,24-27,29,30,32] and only in 4 cases [17,19,28,31] have they speculated about its application in chronic pathological conditions. Thus, most RCT studies have, above all, demonstrated how various technologies used to activate VC are feasible and acceptable and, consequently, how they can have a potential impact on preserving public health.

In relation to the ambitious goal of helping with the implementation of primary prevention, it is obvious that both the limited number of subjects studied and the short time of use of the technological solutions have not yet allowed research to sustain the absolute effectiveness of VC in producing a change in harmful behavior or maintaining good habits that can positively affect people’s health. Conversely, it is known that studies related to lifestyles and how these can be modified in a positive and healthy way require several years of monitoring of a large population. Second, although 6 RCT studies evaluating physical activity and 3 being overweight are present, the comparability of results within each of the two groups is inadequate for differences in average age and sample size of the studied populations, in the types of interface, and in the used outcome measures.

Most of the remaining studies, definable as exploratory, return little information beyond the interest of some authors in evaluating the application of VC in the management of some pathological conditions.

Health promotion can be achieved by using ECA or IVR systems, as the usability and acceptance of these devices is usually high [24,30,46]. They have been used for engaging the elderly in regular physical exercise [24,30,46], and they have been applied in combination with tracking devices and sensors, such as pedometers, to promote a healthy lifestyle in older adults with obesity and a sedentary lifestyle, [20,22,27] and to enhance an adult’s social interaction.

The therapeutic alliance between the patient and the health professional has also been considered an integrative element in all forms of therapy, regarding both the efficacy of treatment and the improvement of adherence. Interestingly, ECAs, especially those exhibiting empathic behaviors, can potentially deliver nonjudgmental support like real face to-face communication, and thus may compensate for clinical failures in some groups of patients. ECA might also be used to promote healthy eating habits specific to neurologic and cardiologic patients. In addition, taking care of nutrition in patients is very important, particularly during pharmacological treatments as many nutrients may interact with drugs (eg, levodopa), thus interfering with their intestinal absorption. Although the ideal characteristics of an ECA developing an empathic relationship with the user are still under study, this interface seems to bring good acceptability in many studies and could be the best solution today in terms of an interface for controlling the regular intake of drugs in patients with chronic diseases.

### Cardiological and Neurological Areas

The review has revealed a certain interest in cardiological and neurological diseases which, as is well known, often require changes in lifestyle, adherence to drug therapies, and specific rehabilitation programs. Specifically, cardiological and neurological diseases are especially relevant for people as they age. They include cardiovascular diseases, such as heart attack and heart failure, cerebrovascular diseases, such as stroke, or neurodegenerative disorders such as PD. Admittedly, these diseases have quite different pathogenic mechanisms and therapeutic treatment, with an acute onset or a chronic course. However, the structure of the rehabilitation programs is quite similar, even if the care plans and their basis might differ at some points. Their main objective is to help the patients in improving their quality of life as much as possible through recovery of their fitness levels and motor and cognitive disabilities. People should be supported in relearning or regaining the ability to deal with their home environment without (extensive) help. Thanks to the progress in acute treatment (eg, coronary angioplasty, thrombolysis, the concept of stroke units, and dysphagia management), most people survive acute pathological events but they are often affected by long-term disability.

Consequently, rehabilitation (mainly as organized inpatient multidisciplinary rehabilitation) remains one of the cornerstones of disability treatment. Rehabilitation strategies help survivors to relearn skills that are lost. In the literature, there are models [67,68] that illustrate disease progression with and without rehabilitation, emphasizing its importance in improving functional performance. The main idea is that rehabilitation is necessary to develop and expand functional recovery in acute disease (eg, heart attack or stroke) and to slow down cognitive and motor function decline in chronic disease (eg, heart failure or PD). Currently, the adjustment of chronic drug therapy and rehabilitation therapy may start from the acute care hospital after the person’s overall condition has been stabilized, and it should be a long ongoing process continued after being out of clinic for months or years. However, the surveillance of correct drug intake and the continuation of rehabilitation treatment are difficult in the domestic environment, especially if not supported adequately by informal caregivers and in the presence of frailty conditions likely to be relevant for the older population. So, in most cases, the correct drug intake may not occur and the rehabilitation process will end following clinical discharge, so patients will have a risk of a clinically unstable condition or a gradual increase in their disability level by detraining. In this scenario, the intervention of a VC would have significant and positive implications in terms of home therapeutic management of patients suffering from these diseases.

### Lack of Continuity of Care

The lack of continuity of care leads to a gap between the real patient’s condition and the level of recovery that they could reach with a constant, well-structured rehabilitation process [51,52]. To maximize the rehabilitation outcome, the patient’s motivation should be sustained during exercise repetition. Patients need to be able to practice motor actions in different tasks and environmental contexts to develop motor schemata that are versatile enough to meet the situations they encounter in daily life. The difficulty level of the motor and cognitive task assigned to the patient, together with their subjective awareness of the obtained global performance and the quantity and quality of feedback received during training, can influence patient motivation and produce different means of acting and different performances.

In addition to improving motor and cognitive manifestations by proper monitoring and rehabilitation programs, one of the main elements determining quality of life is related to rehabilitation strategies oriented to promote independence in basic and instrumental activities of daily living. For all these reasons, it is time to design and implement coaching systems capable of activating the appropriate strategies to improve the motor and cognitive disabilities of patients, including those promoting independence in personal hygiene, dressing, self-feeding, using the telephone or other forms of communication, and taking prescribed medications. Thus, continuously applied and personalized rehabilitation programs help people with disabilities to improve the body functions that limit reinsertion into their home or community, living independently, and participating in education, the labor market, and civic life [41]. Currently, the major disadvantages of home-based rehabilitation solutions are the lack of specialized equipment and insufficient alignment and adaptability of the technical possibilities to the individual care needs and abilities, but *ad hoc* VC systems could be the answer to these problems.

### Future Perspective of Virtual Coaching System

The review of the VC literature confirms an increased diffusion of technologies with strong interaction with human beings in the world of health and medicine. The increase in interest on this issue is evident not only in the number of publications since the beginning of the current year but also in the guiding vision of the EU’s Horizon 2020 research framework program.

If up to now most studies have tested the goodness of the technological interlocutor and the availability of the human one, in the future, we will try to strengthen this relationship in the medical sector as we are working to foster patients’ engagement toward a process of self-awareness and self-confidence in health management. Nevertheless, advanced modes of presence will lead to ubiquity of the VC and allow further interaction modes. Integrating the VC within the real world in terms of an augmented reality (eg, the avatar could also be present in the car) will broaden its abilities to guide the user and therefore increase the literacy of the suggested actions. Advanced sensing and acting levels allow seamless adaptation in relation to the people’s conditions. Prospectively, multiple linked coaches will allow better monitoring and guidance of the user. Furthermore, the to-be virtual coach will not only improve basic treatment scenarios, but the advanced AI may also infer new treatment strategies.

Hopefully, these changes will allow us to deal with increasingly complex clinical situations, mainly belonging to the world of chronic diseases and disabilities. The lesson acquired so far, aimed at promoting coaching interventions often on healthy subjects, independent thinking, and acting, represents an important step in transferring the experience of the interventions on the major themes of primary prevention to the more complicated and specific ones of secondary prevention.

Thus, the future of VC for frail or sick people is expected to provide solid evidence about the usefulness and effectiveness in preserving physical, cognitive, mental, and social well-being for as long as possible. More specifically, the VC will educate and finally empower patients to increase their adherence to drug therapy and to pursue physical and cognitive rehabilitation programs to regain independence with a healthier lifestyle and a better quality of life.

### Strengths and Limitations of This Scoping Review

This scoping review was guided by a protocol reviewed by a research team that adopted Arksey and O’Malley’s definition for scoping reviews at the outset of the study and found that their simple definition was generally useful in guiding study selection. To ensure a broad search of the literature, the search strategy included 3 electronic bibliographic databases, the reference list of different articles, the websites of relevant journals, and the snowball technique. Each citation and article were then reviewed by 3 independent reviewers belonging to different disciplines (medicine and bioengineering) who met at regular intervals to resolve conflicts.

For several reasons, this review brings new information to the reader’s attention. First, it revised the definition of virtual coach, demonstrating the current validity of the first description made by Ding and how it can still include the technological developments of recent years aimed at improving interaction between virtual coach and user. Second, with an innovative approach, the review significantly aimed at highlighting which medical care sectors were considered to apply VC. Considering this extensive analysis, the review aimed to return useful information to bioengineers regarding the current potentialities of the medical application of VC and look at its possible future uses.

Nevertheless, some limitations are worth noting. First, because of variability in the conduct of scoping reviews, we are aware that there is a need for methodological standardization to ensure the strength of evidence. Second, this review may not have identified all the papers in the published literature despite attempts to be as comprehensive as possible. Our search algorithm included 9 different terms (and their declination) previously used to describe the VC devices and services; however, other terms may also exist. Although our search included 3 databases (ie, PubMed, Scopus, and Embase), the overall search strategy may have been biased toward health and sciences. Moreover, we may have missed some papers in the gray literature.

### Conclusions

The continuous technological progress is changing the impact on users of the use of a humanoid avatar, which is an artificial figure apparently substitutive for a health professional. This relationship could entail numerous preconceptions, such as the lack of a direct dialogue between doctor and patient. As already underlined, a trusty relationship will be based on the good interaction between the user and the avatar and on how much the latter is recognized as an element of a high-quality medical care process. However, the psychological implications of this relationship can only be known through the concrete application of these technologies. Based on our analysis, we must look forward with confidence, aware that there are still several open questions. In a broader vision, many articles on VC have directly or indirectly addressed medical aspects of utmost importance in both primary (eg, risk factors reduction) and secondary (eg, treatment adherence) prevention.

However, the review gained evidence that VC is more rarely applied in medical care for secondary prevention. As a matter of fact, although physical activity is mostly encouraged by a VC system, we know that it cannot be compared with a real rehabilitation program, whose indication is of extreme importance in patients suffering from acute disabling events or motor functional impairments because of several chronic conditions. Therefore, in the current scenario of VC application, rehabilitation is the great absentee. Thus, looking at a secondary prevention of chronic diseases, VC has a concrete potential to improve medication adherence and to extend existing rehabilitation programs for patients, providing a great opportunity to guarantee the continuity of care between hospital and home. Considering these considerations, it is essential that the constructive dialogue between bioengineers and clinicians in creating innovative technological platforms continues and improves to deal synergistically with new challenges in the world of medical care.

## Abbreviations

AI: artificial intelligence
COPD: chronic obstructive pulmonary disease
ECA: embodied conversational agent
e-coach: electronic coach
eCoaching: electronic coaching
eHealth: electronic health
e-learning: electronic learning
EU: European Union
IVR: interactive voice response
PD: Parkinson disease
RCT: randomized controlled trial
VC: virtual coaching
VNA: virtual nursing avatars
